# The discovery of a new type of innervation in lymphoid organs

**DOI:** 10.14814/phy2.15604

**Published:** 2023-02-23

**Authors:** Kaiyun Wu, Ruixi Li, Yanlin Zhang, YanMei Liu, MinChen Wang, Jinyu Huang, Changlai Zhu, Jianping Zhang, Xiangshan Yuan, Qingqing Liu

**Affiliations:** ^1^ Department of Anatomy Medical College of Soochow University Suzhou China; ^2^ Department of Anatomy, Histology and Embryology Shanghai Medical College of Fudan University Shanghai China; ^3^ Department of Neurology Second Affiliated Hospital of Soochow University Suzhou China; ^4^ Jiangsu Key Laboratory of Neuroregeneration Nantong University Nantong China

**Keywords:** anterograde tracing, innervation, Langerhans cells, lymph organs, three‐dimensional reconstruction

## Abstract

It is well known that the main forms of innervation are synapses and free nerve endings, while other forms of innervation have not been reported. Here, we explore a new way of innervating lymphoid organs. Male Sprague‐Dawley rats were used for studying the innervation of sympathetic nerve fibers in lymph nodes by means of anterograde tracking, immunoelectron microscopy, three‐dimension reconstruction analysis, and immunofluorescence labeling. The results showed that the Fluoro‐Ruby labeled nerve endings targeted only a group of cells in the lymph nodes and entered the cells through the plasma membrane. The electron microscopy showed that the biotinylated glucan amine reaction elements were distributed in the cytoplasm, and most of the biotinylated glucan amine active elements were concentrated on the microtubule and microfilament walls. Birbeck particles with rod‐shaped and/or tennis racket like structures can be seen in the labeled cells at high magnification, and Birbeck particles contain biotinylated glucan amine‐reactive elements. The immunofluoresence results showed that the Fluoro‐Ruby‐labeled nerve innervating cells expressed CD207 and CD1a protein. This result confirmed that the labeled cells were Langerhans cells. Our findings suggested that Langerhans cells might serve as a “bridge cell” for neuroimmune cross‐talking in lymph organs, which play an important role in transmitting signals of the nervous system to immune system. This study also opened up a new way for further study of immune regulation mechanism.

## Abstract 2

We report a new type of innervation in lymphoid organs. Our findings suggested that LC might serve as a “bridge cell” for neuroimmune cross‐talking in lymph organs, which play an important role in transmitting signals of the nervous system to immune system.

## 1 INTRODUCTION

In 1868, Langerhans discovered a dendritic cell colony in the human epidermis, which is now named after him (Langerhans, 1868). At that time, he was convinced that the cutaneous nerve endings were found by gold chloride staining. However, the appearance of electron microscope finally overturned the “neural” theory of Langerhans cell (LCs) (Ferreira‐Marques, 1951; Niebauer & Sekido, 1965). After the mid‐1970s, a series of observations made us know that these cells are bone marrow‐derived antigen‐presenting dendritic cells (DC) (Katz et al., 1979; Romani et al., 2012), which are located in the epithelium, spleen, thymus, and lymph nodes, which has fundamentally changed our understanding of LC biology (Hoshino et al., 1970; Jimbow et al., 1969; Kubo et al., 2009; Vernon et al., 1973). The morphological characteristics of LCs are that there are unique rod‐shaped or tennis racket shaped Birbeck particles (BGs) in the cells (Birbeck et al., 1961). Recent studies have shown that BGs are not an endocytic structure, but a subdomain that forms the endoplasmic recirculation chamber of langerin aggregation. Langerin can act as a receptor to capture specific microbial antigens through this unique antigen‐presenting cell population (Birbeck et al., 1961; Jimbow et al., 1969; McDermott et al., 2002). The LC phenotypic marker is the expression of cd207 and CD1a (Kelly et al., 2016; Raaby et al., 2017; Valladeau et al., 2002).

It is well known that lymphoid organs are innervated by sympathetic nerves (Elenkov et al., 2000). However, the form of sympathetic innervating immune cells and the mechanism of transmitting signals to immune cells are not clear (Nance & Sanders, 2007; Veres et al., 2009). Our previous study found that the sympathetic nerve only innervated S100^+^ immunoreactive cells in lymph nodes (Huang et al., 2013), not in the form of free nerve endings. These S100^+^ immunoreactive cells were mononuclear, polymorphous, and light nuclear density large cells, which also conformed to the morphological characteristics of LCs. In order to find out whether LC is a “bridge cell” of neuroimmune interaction between the nervous system and the immune system, we used Fluoro‐Ruby (FR) and biotinylated dextran amine (BDA) as anterograde tracers to track the sympathetic nerve endings, to determine whether the sympathetic nerve in the lymphatic tissue only dominates LCs and their innervation forms, so as to reveal the true nature of its innervation. This qualitative study will provide a morphological basis for the study of neuroimmune regulation mechanism.

## 2 METHODS

### 2.1 Experimental animals

Male Sprague‐Dawley (SD) rats weighing 250–280 g were used in this study and purchased from the experimental animal center of Suzhou University (Suzhou, China). These experiments have been approved by the ethics committee of Suzhou University, and all procedures have been carried out in accordance with the arrive guidelines and the laws, guidelines and policies of China on animal experiment, housing and nursing. All experimental rats used in this study were killed by cardiac perfusion with 4% paraformaldehyde (under deep anesthesia, see the following experimental methods for details).

### 2.2 Tracing and tissue sectioning

Male SD rats were used to trace the sympathetic nerve fiber endings innervating the lymph nodes. After anesthetizing the rats with pentobarbital sodium (20 mg/kg, i.p.), a total volume of 1 μL 30% FR (fluorochrome, Inc., USA) or 1 μL of 15% biotinylated glucan amine (BDA) was injected into the superior cervical sympathetic ganglia. In the experimental group, 25 cases were injected (except injection failure). The control group (12 cases), in order to prove that FR‐labeled fibers are caused by axonal transport rather than simple free diffusion, we injected the same amount of 30% FR into the position of superior cervical ganglion after resection. After 14 days of survival, the rats were anesthetized with pentobarbital sodium (20 mg/kg i.p.) and perfused with 4% paraformaldehyde prepared with 0.1 M phosphate buffer. The 4–5 ipsilateral cervical lymph nodes were fixed with the same fixative overnight and then cryopreserved with 50% sucrose. The slices (25 μm) were cut transversely on a frozen microtome, mounted on gelatin coated slides, and observed under a laser confocal microscope.

### 2.3 Immunoelectron microscope

The cervical lymph nodes of experimental rats were cut with a vibrating microtome (Leica vt10 00s) for 40 μm thick slice. The tissue sections were incubated with Streptococcus avidin biotin complex (SABC, 1:200) for 40 min, reacted with DAB solution for 5 min, then permeated with 2% osmium tetroxide, dehydrated with gradient ethanol, and embedded with EPON 812 (FLUKA). For transmission electron microscope slicing, 70 nm thick ultrathin sections were cut with a diamond knife (Diatom Co., Ltd.). It was stained with uranyl acetate and lead citrate and observed under Philips CM‐10 transmission electron microscope (TCS SP5, Leica).

### 2.4 Immunofluorescence

The slides were, respectively, incubated with primary rabbit antibodies against CD207 and CD1a diluted in PBS (1:250), overnight at 4°C, followed by incubation with FITC‐conjugated goat anti‐rabbit IgG (1:500) for 50 min at room temperature. The slides were rinsed in 0.01 M PBS three times between steps and then mounted with mounting medium. Finally, the slides were observed and photographed under a fluorescence microscope.

### 2.5 Gold chloride staining

The staining was performed according to Gairn's technic as described by Becker and Zimmermann (1955). Specimens of full thickness of the lymph nodes were placed in a mixture of pure formic acid and filtered lemon juice (1:3) in the dark for 10–30 min. The specimens were then removed from the solution, transferred to 1% aqueous solution of gold chloride in the dark for another 10–30 min. Finally, the specimens of the lymph nodes were transferred to a 25% aqueous solution of formic acid in the dark for 24 h, and then dehydrate, sealed, and observed under microscope.

## 3 RESULTS

**FIGURE 1:**
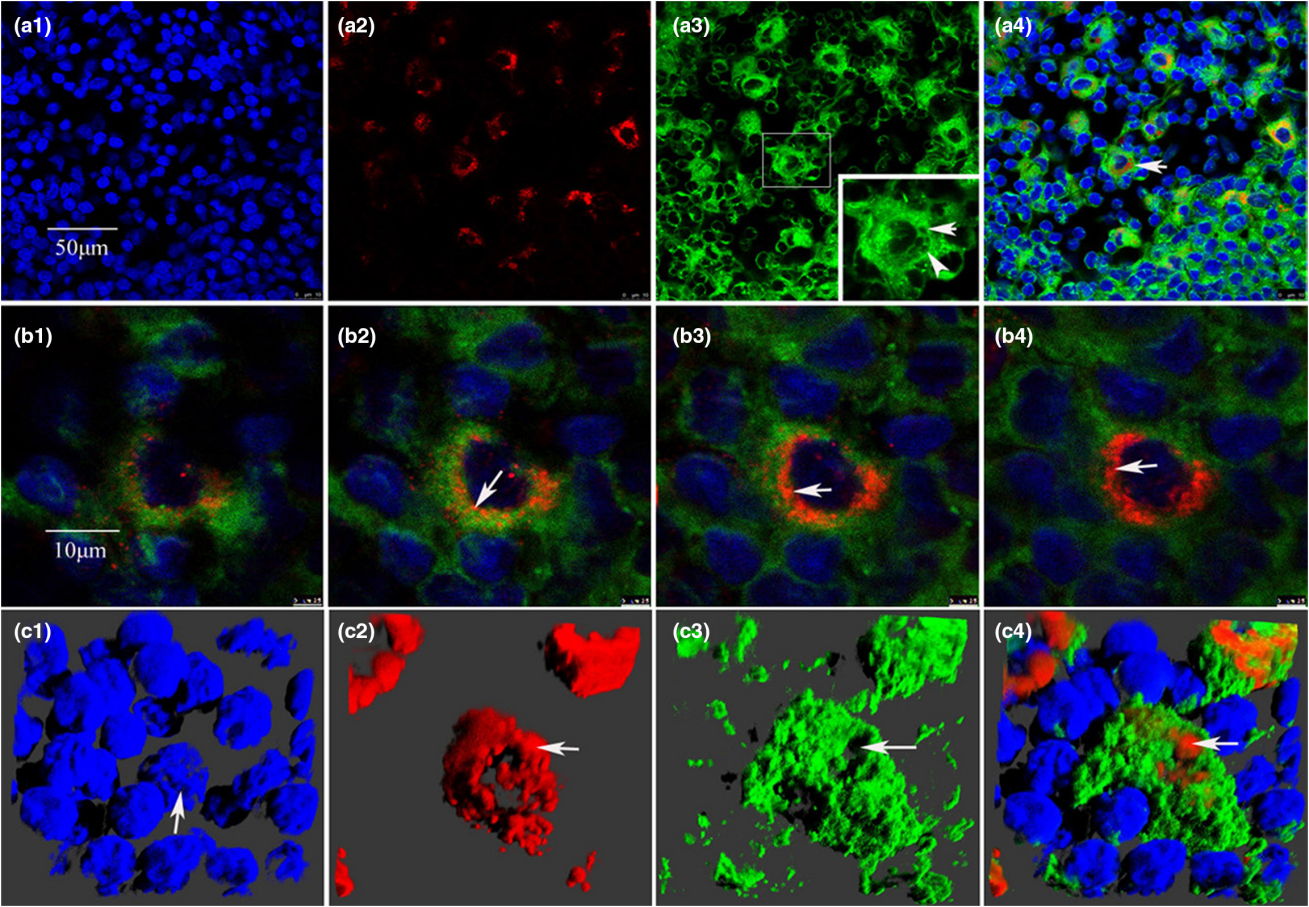
Distribution of FR‐labeled sympathetic nerve endings in lymph nodes. (a1) DAPI nuclear staining; (a2) FR‐labeled nerve fiber terminals; (a3) DIO cell membrane staining; (a4) merge images. From (a1)–(a4), it can be seen that the FR‐labeled nerve fiber terminals only target specific large cells. Holes in the cell membrane can be seen in (a3), which is the path of nerve fibers through the cell membrane (white arrow). (b1)–(b4) shows that FR‐labeled nerve fiber terminals penetrate the cell membrane and enter the cell (white arrow). (c1)–(c4) showed three‐dimensional reconstruction, and the RF‐labeled nerve fibers were located in the cells.

**FIGURE 2:**
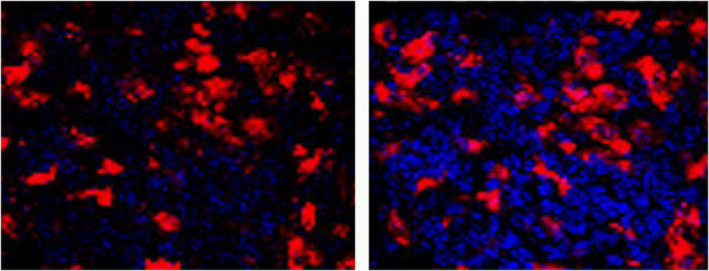
Distribution of injected the same amount FR into the position of superior cervical ganglion after resection (control group). The results showed that the FR signal in the control group was irregularly distributed in the intercellular diffusion and did not surround specific nuclei.

**FIGURE 3:**
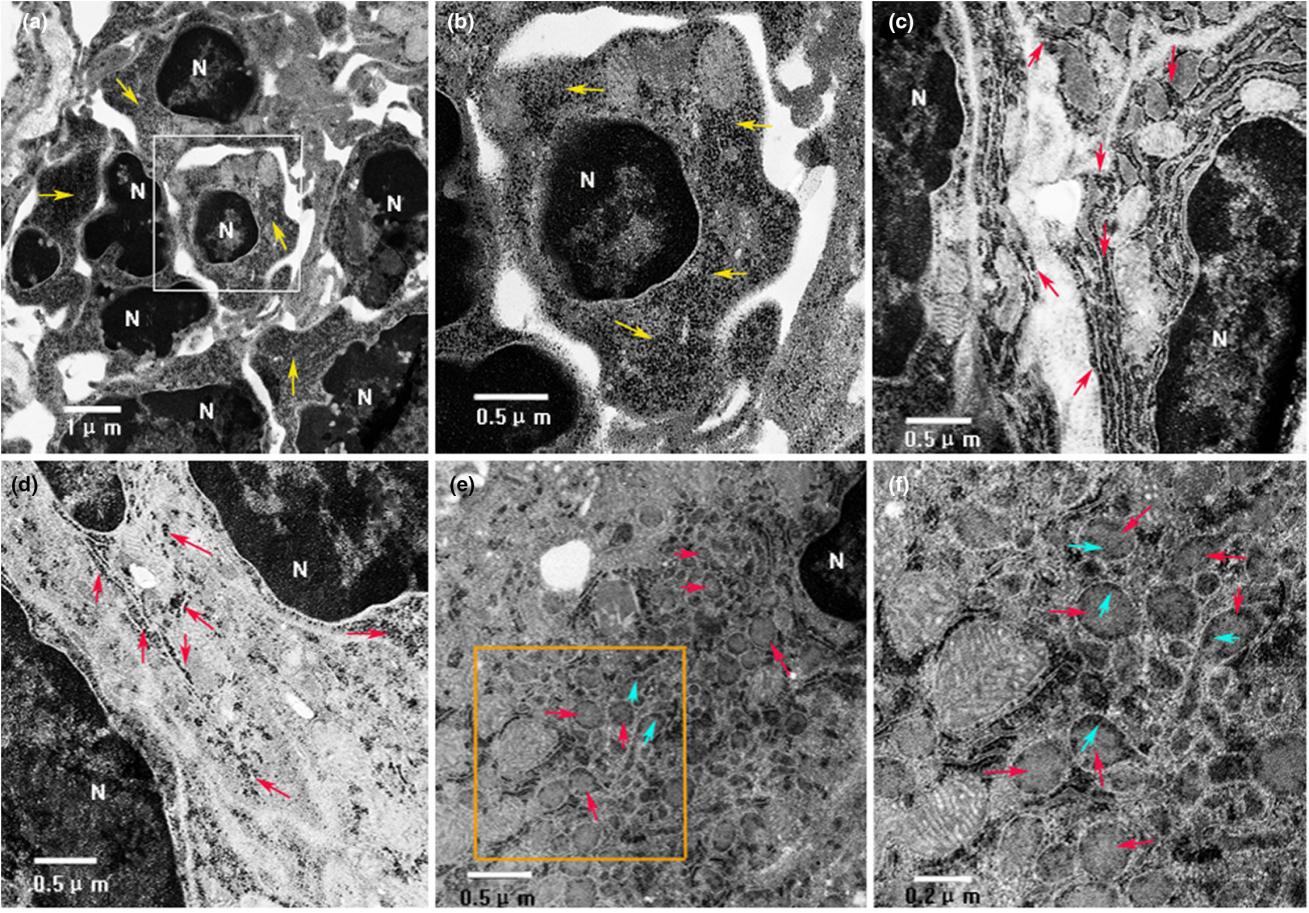
Electron microscope shows the distribution and location of BDA‐labeled sympathetic nerve endings in LCs. In (a) and (b), BDA reaction elements are distributed throughout the cytoplasm (yellow arrows). BDA active elements are distributed along the walls of microtubules and microfilaments (c and d red arrows). BGs formed by rod‐shaped and/or racquet like structures can be seen under the high‐power microscope of labeled cells, and BDA response elements are distributed (e and f red arrows). The results of electron microscopy suggest that BDA response elements are distributed along microtubule and microfilament walls and enter BGs (e and f green arrows). BGs may be an intracellular neural signal conversion device.

**FIGURE 4:**
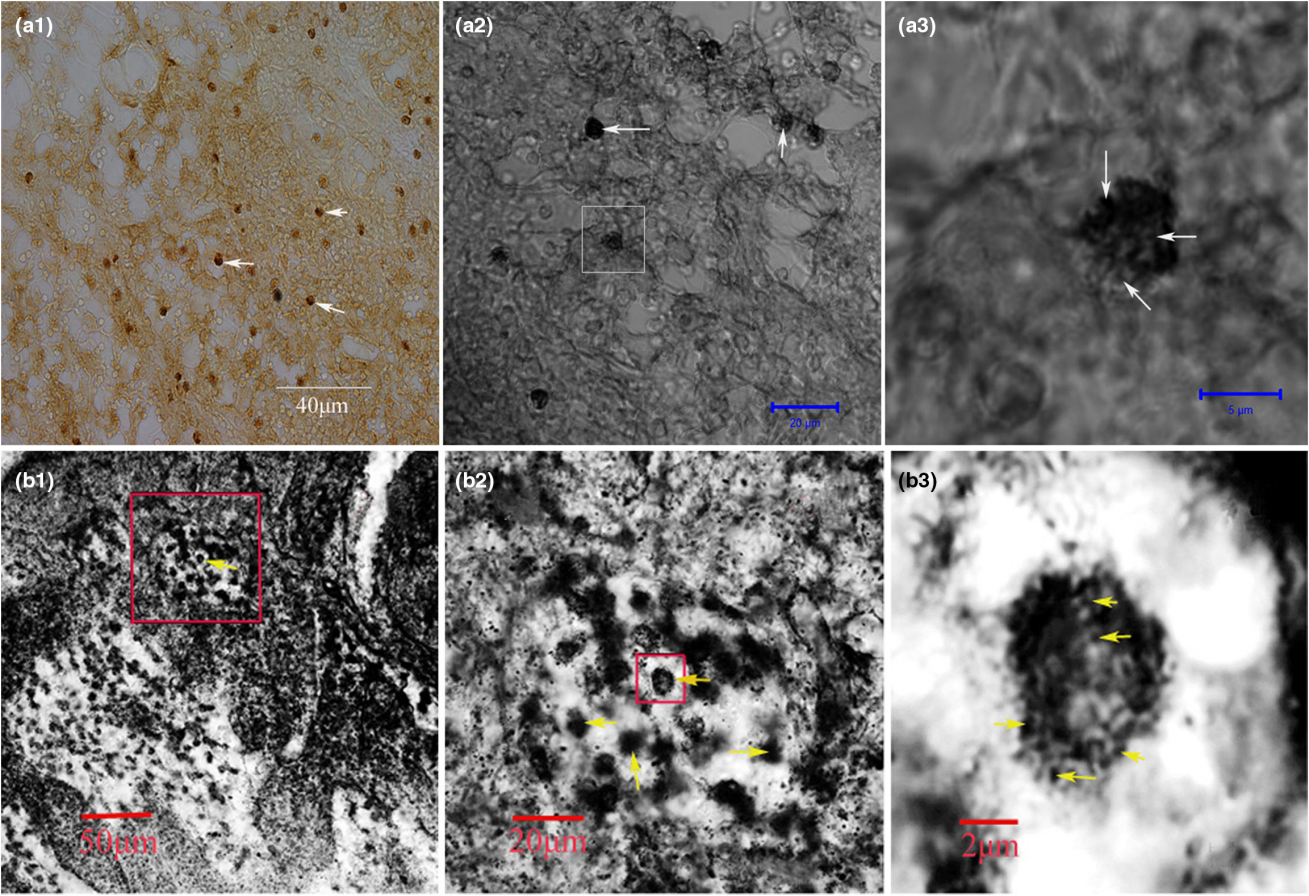
Distribution of BDA‐labeled cells in lymph nodes (a1–a3) and gold chloride stained cells in lymph nodes (b1–b3). BDA‐labeled lymph nodes were stained with immunocytochemistry (SABC) and observed under confocal microscope. Positive cells (a1 and a2 white arrows) were found in BDA‐labeled lymph nodes, and BDA‐labeled nerve fibers were clearly located in cells (a3 white arrows) under high‐resolution laser confocal microscope. The gold chloride stained cells were mainly distributed in the paracortical and medullary areas (b1 and b2) of lymph nodes. Under high‐power confocal microscope, these nerve fiber endings stained with gold chloride were clearly visible (b3 yellow arrow).

We performed anterograde tracing by injecting the tracer Fluoro‐Ruby (FR) into the superior ganglion of the rat cervical sympathetic trunk to mark the distribution of sympathetic nerve endings in the lymph nodes. The lymph node tissues were sectioned and observed under the fluorescence microscope, and the cell membrane was stained with fluorescent dye DIO for three‐dimensional reconstruction. The results showed that the labeled nerve endings could be seen in all 25 rats successfully injected with FR, and the FR‐labeled nerve endings targeted only a group of cells in the lymph nodes and entered the cells through the plasma membrane (Figure 1a1–a4 and b1–b4). 3D reconstruction further demonstrated that the labeled nerve fibers were indeed located in the cells (Figure 1c1–c4 and Supplementary movie). In order to prove that FR markers are caused by axonal transport rather than simple free diffusion, we injected the same amount of FR into the position of superior cervical ganglion resection in the control group to observe the distribution of FR markers. In the 12 cases of control group, FR markers were distributed irregularly and freely among cells (Figure 2), and did not surround the nuclei of specific cells in lymph nodes as in the experimental group. The comparison between the experimental group and the control group showed that the labeled nerve fibers did come from axonal transport rather than free diffusion. At the subcellular level, we further used BDA as an anterograde tracer to track and label sympathetic nerve endings. The results of electron microscopy showed that the biotinylated glucan amine (BDA) reaction elements of BDA tracer were distributed in the cytoplasm (Figure 3a,b Yellow arrowhead), and most of the BDA active elements were concentrated on the microtubule and microfilament walls (Figure 3c,d Red arrows). Birbeck particles (BGs) with rod‐shaped and/or tennis racket like structures can be seen in the labeled cells at high magnification (Figure 3e,f Red arrows), and BGs contain BDA‐reactive elements (Figure 3f Gree arrows). BG structure only exists in LCs, suggesting that the labeled cells may be LCs. In addition, BDA‐labeled immunofluorescence staining also confirmed that BDA‐labeled nerve fibers were indeed present in the cells (Figure 4a1–a3 White arrowhead). The white arrow (Figure 4a3) represents the nerve fiber structure.

**FIGURE 5:**
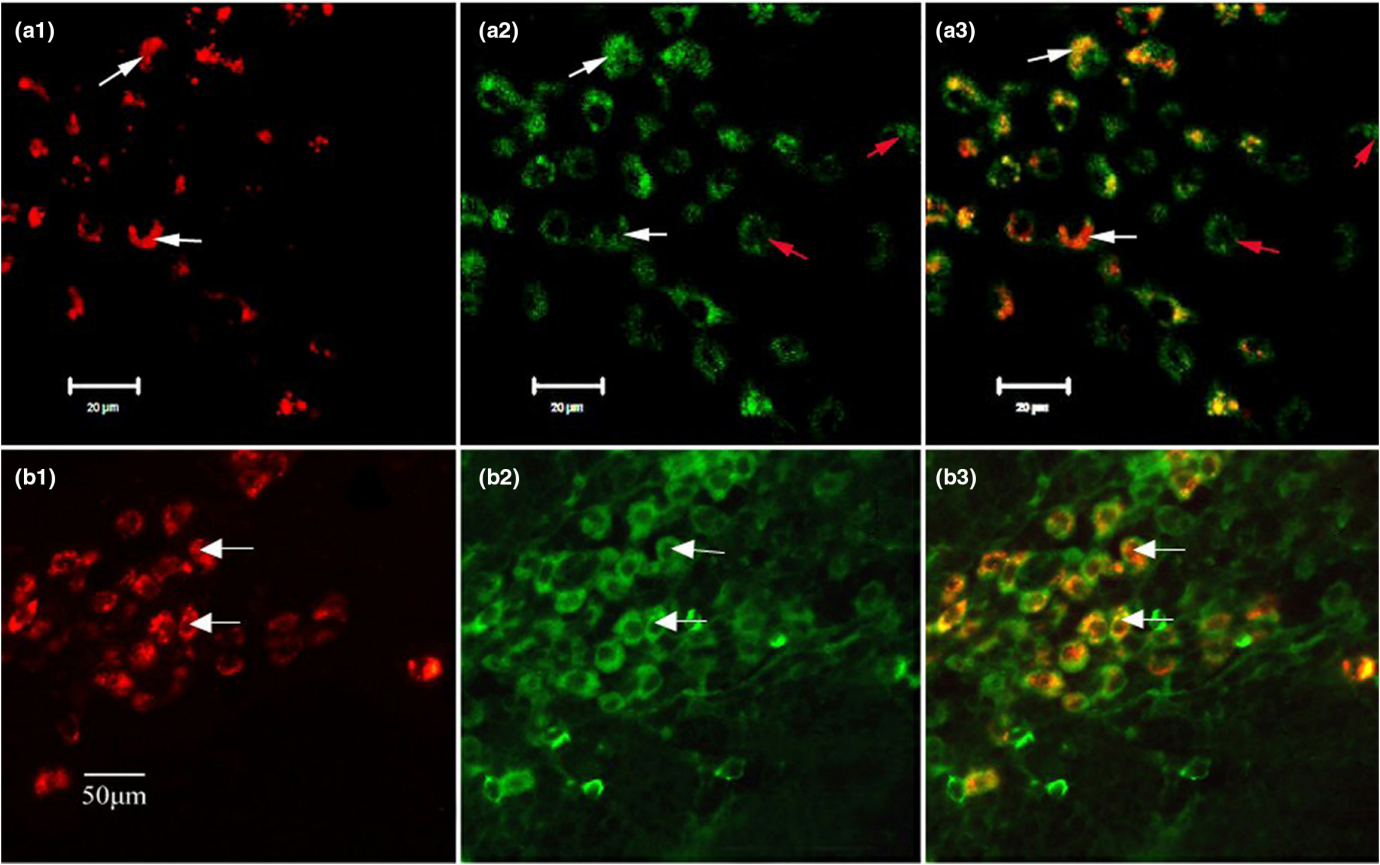
Immunofluorescence showing the expression of CD207 and CD1a in FR‐labeled cells (a1, b1) are FR‐labeled nerve fibers. (a2, b2) were CD207 and CD1a positive cells, respectively. FR‐labeled lymph nodes were stained with monoclonal anti‐CD207 and CD1a antibodies, respectively. The results showed that the labeled cells were LCs, because CD207 and CD1a were specific markers of LCs. In (a2) and (a3), white arrows indicate positive cells consistent with labeled cells, and red arrows indicate unlabeled positive cells.

In order to further determine whether the labeled cells were LCs, the second group of experiments used specific antigens of LCs (cd207 and CD1a) for immunofluorescence staining. Immunofluorescence showed that the FR‐labeled innervated cells expressed CD207 (Figure 5a1–a3) and CD1a (Figure 5b1–b3) proteins. This result confirmed that the labeled cells were LCs.

We repeated Langerhans' staining experiment and stained rat lymph nodes with gold chloride. In an attempt to clarify some previous doubts about LCs, LCs is not a nerve cell, why can LCs be stained with gold chloride? Our results show that typical gold chloride staining positive cells can be seen in rat lymph nodes (Figure 4b1–b3). Using high‐resolution laser confocal microscope analysis, it was proved that the gold stained structure was the nerve fiber terminals (Figure 4b3 Yellow arrowhead), not the cells themselves. This result helps us better understand why LCs (non‐nerve cells) can be stained with gold chloride.

## 4 DISCUSSION

More and more evidence shows that the communication between the nervous system and the immune system is crucial to the changes of immune function (Andersson & Tracey, 2012; Kerschensteiner et al., 2009). At present, the known neuroimmune regulatory pathways mainly include neuroendocrine pathway and sympathetic innervation. The interaction between nerve and immune cells plays an important role in cellular and humoral mediated immune responses (Dustin, 2012). Recently, immunofluorescence staining has been used to study the distribution of nerve fibers in mouse lymph nodes and the combination of nervous system and immune cells (Hu et al., 2019). Other studies have shown that antigen‐presenting cells (APCs) have abundant innervation in the T‐cell rich region, subnuclear layer, and extrafollicular cortex of rat lymph nodes (Wülfing & Günther, 2015). APCs belong to dendritic cells, suggesting that dendritic cells play an important role in immune response. This study proved for the first time that nerve fibers from sympathetic nerves only innervate LCs in lymph nodes and directly target cells. Previously, it was thought that the synapse between nerve and target organ was mainly in the form of synapse (Doebel et al., 2017). This direct targeting intracellular form has never been seen before (Guillery, 2005). This finding challenges previous ideas in neuroscience. LCs is a kind of dendritic cells. There is evidence that the immune response induced by LCs is complex and depends on the characteristics of antigens and the localization of stimulants (Doebel et al., 2017; Jimbow et al., 1969).Our qualitative study confirmed that the sympathetic nerve in lymphoid tissue only innervates LC, and found a new form of innervation, which also provides more convincing evidence for the important role of dendritic cells in the immune response. However, why should lymphoid organs be innervated in this way? Although the function and biological significance of this innervation mode cannot be explained at present, we observed that BDA‐labeled microtubules, microfilaments and BGs forming rod or tennis racket in the target cells by immunoelectron microscopy. These BDA response elements make us believe that the nerve signal substances may be conducted along microtubules and microfilaments, and Birbeck particles may be the nerve signal conversion devices in cells. We can assume that when our body encounters various antigens, neuromodulators will be released from different subdomains of microtubule and microfilament networks in LCs, which will enable LCs to process antigens and antigen presentation quickly and accurately. But its real mechanism needs further study.

We also want to clarify some questions and puzzles about LCs. It is well known that gold chloride staining is a specific staining of nerve tissue and cells. However, LCs are not nerve cells. Why are LCS stained with gold chloride? It was a fact that LCs could be stained with gold chloride, which also caused controversy at that time. This doubt has not been reasonably explained so far. The results of this study showed that the nerve fibers innervating LCs directly entered the cells. Is this because the nerve fibers of LCs are stained with gold chloride? Therefore, we repeated Langerhans' staining experiment and stained rat lymph nodes with gold chloride. Results typical gold staining positive cells were found in lymph nodes. Using high‐resolution laser confocal microscope analysis, it was proved that the gold stained structure was the nerve fiber terminals, not the cells themselves. This result well explains why LCs (non‐nerve cells) can be stained with gold chloride (Schmued, 1990). The doubts of this century are well explained. We believe that LCs act as a “dialogue cell” of nerve immune communication in lymphoid organs and play an important role in transmitting nerve signals to the immune system. This study also opened up a new way for further study of immune regulation mechanism.

## AUTHOR CONTRIBUTIONS

Kaiyun Wu designed and supervised whole study and performed major experiment, including the details of labeling and immunoelectron microscopy, analyzed the data, and prepared the manuscript. Ruixi Li contributed the key technologies of immunoelectron microscopy. Yanlin Zhang, YanMei Liu, MinChen Wang and Jinyu Huang performed the anterograde labeling, immunohistochemistrical experiments and participated in image processing. Changlai Zhu, Jianping Zhang, Xiangshan Yuan and Qingqing Liu contributed the experimental preparations and pictures of immunoelectron microscopy. All authors discussed the results, provided comments and reviewed the manuscript.

## CONFLICT OF INTEREST STATEMENT

The authors declare no competing financial interests.

## ETHICS STATEMENT

These experiments have been approved by the ethics committee of Suzhou University, and all procedures have been carried out in accordance with the arrive guidelines and the laws, guidelines and policies of China on animal experiment, housing and nursing.

## Supporting information


Movie S1
